# Supplementary material for: The genome sequence of the Dalmatian wall lizard, Podarcis melisellensis (Braun, 1877)
Source: Wellcome Open Res. 2025 May 19;10:247. [Version 1] doi: 10.12688/wellcomeopenres.24140.1 (PMC12186021; doi:10.12688/wellcomeopenres.24140.1)
Supplement: Peer review history

# Reviewer report: Reviewer response for version 1

**DOI:** 10.21956/wellcomeopenres.26631.r124747

**Published:** 2025-06-25

**Authors:** Sunandan Das

**Affiliations:** 1 University of Helsinki, Helsinki, Finland

The article is a thorough data note reporting the reference genome for *Podarcis melisellensis*. Authors have used long read and chromatin conformation capture sequencing to generate the genome sequence assembled at chromosome level. The methodology is detailed and exact wet lab and *in silico* protocols (including the software name) for everything reported in the Genome sequence report section can be traced in the methods without ambiguity. Methods and protocols are sufficiently detailed to allow the reproduction of this work. *Podarcis* spp. usually possess 38 chromosomes (2n), including the sex chromosomes. So, the number of chromosome pseudomolecules generated with conformation capture seq correctly adds up. The genome size is also around the value known for this genus. The assembly parameters and statistics are of sufficient quality (it achieves the general standard in recent reptile reference genome papers). *Podarcis* spp. is an interesting, predominantly mediterranean lacertid radiation from speciation, population genomic, systematic and comparative genomic points of view and this data note reports a reference genome that will be valuable in all such studies. The only change I would make is to change 'Least Concerned' in the Background section to Least Concern as that is the more standard usage. The usage in the paper, however, is nothing wrong and authors can ignore this if they so wish.

Are sufficient details of methods and materials provided to allow replication by others?

Yes

Is the rationale for creating the dataset(s) clearly described?

Yes

Are the datasets clearly presented in a useable and accessible format?

Yes

Are the protocols appropriate and is the work technically sound?

Yes

Reviewer Expertise:

Herpetology, Phylogenomics, Speciation genomics, Systematics

I confirm that I have read this submission and believe that I have an appropriate level of expertise to confirm that it is of an acceptable scientific standard.

# Reviewer report: Reviewer response for version 1

**DOI:** 10.21956/wellcomeopenres.26631.r124748

**Published:** 2025-06-23

**Authors:** Hong-Xin Xie

**Affiliations:** 1 University of Glasgow, Glasgow, Scotland, UK

The authors present new genome assemblies for a wall lizard *Podarcis melisellensis*. The genome is chromosome level, curated and quality assessed in a well established pipeline. The reference genome generated will provide a valuable resources for genetic study of the species and comparative genomic studies in wall lizards and other squamate reptiles as well. The only concern I have about the reference genome is the relatively "low" quality of the assembly (contig N50 ~3 mb and 94.94% of sequences assigned to chromosomes). These statistics are generally good, however, considering the study used 41.0x coverage of Pacbio HiFi reads (> 30x is generally considered good) and more than 100x Hi-C data for scaffolding, the assembly matrix  seem not good enough. Recent genomes using HiFi +Hi-C can easily reach contigs spanning almost the whole chromosome and a scaffold assignment rate more than 95%. A  relatively low quality of the assembly could be caused by species specific factors or the quality of the raw sequencing reads. The first one is less likely because other wall lizards using similar strategy can reach much higher assembly matrix (like Podarcis raffonei GCA_027172095.1). Adding a report about the raw HiFi read quality may help explain the assembly matrix. Using tail tip stored in ethanol may not yield ideal high molecular weight DNA but is totally understandable because this is non-lethal sampling. Secondly, from figure 5 the Hi-C interaction plot, there seems to be some mis-assemblies, like 9th to 11th chromosome that have clear boundaries within a chromosome. Maybe a slightly more detailed error correction report can help explain the result.

Are sufficient details of methods and materials provided to allow replication by others?

Yes

Is the rationale for creating the dataset(s) clearly described?

Yes

Are the datasets clearly presented in a useable and accessible format?

Yes

Are the protocols appropriate and is the work technically sound?

Yes

Reviewer Expertise:

genomics, evolutionary genomic, population genomics

I confirm that I have read this submission and believe that I have an appropriate level of expertise to confirm that it is of an acceptable scientific standard.

# Reviewer report: Reviewer response for version 1

**DOI:** 10.21956/wellcomeopenres.26631.r124755

**Published:** 2025-06-23

**Authors:** Mark Wilkinson

**Affiliations:** 1 The Natural History Museum, London, UK

This is a report of the genome sequence of a Dalmatian wall lizard, a widely distributed lacertid with many island populations, numerous named subspecies and is thus of interest for studies of speciation and local adaptation. The study is of high quality. I was pleased to see the precise locality and subspecific identity of the voucher included in the report.  As a taxonomist I would recommend some very minor changes to how taxon names are presented.  On first use in Abstract and Mani text I would use the full name "Podarcis melisellensis (Braun, 1877)" as in the title.  It doesn't matter if the parenthesis in the authority part of the species name is immediately followed by another '(Dalmation wall lizard...). Secondly on first use in isolation in the text 'The species is one of....'  I would add the authority for the genus name Podarcis Wagler, 1830.

Are sufficient details of methods and materials provided to allow replication by others?

Yes

Is the rationale for creating the dataset(s) clearly described?

Yes

Are the datasets clearly presented in a useable and accessible format?

Yes

Are the protocols appropriate and is the work technically sound?

Yes

Reviewer Expertise:

Herpetology, Phylogenetics

I confirm that I have read this submission and believe that I have an appropriate level of expertise to confirm that it is of an acceptable scientific standard.
